# Cytochrome P450 3A Enzymes Are Key Contributors for Hepatic Metabolism of Bufotalin, a Natural Constitute in Chinese Medicine Chansu

**DOI:** 10.3389/fphar.2019.00052

**Published:** 2019-02-04

**Authors:** Zi-Ru Dai, Jing Ning, Gui-Bo Sun, Ping Wang, Feng Zhang, Hong-Ying Ma, Li-Wei Zou, Jie Hou, Jing-Jing Wu, Guang-Bo Ge, Xiao-Bo Sun, Ling Yang

**Affiliations:** ^1^Key Laboratory of Bioactive Substances and Resources Utilization of Chinese Herbal Medicine, Ministry of Education, Institute of Medicinal Plant Development, Chinese Academy of Medical Sciences and Peking Union Medical College, Beijing, China; ^2^College of Pharmacy, Dalian Medical University, Dalian, China; ^3^Institute of Interdisciplinary Integrative Medicine Research, Shanghai University of Traditional Chinese Medicine, Shanghai, China; ^4^Dalian Institute of Chemical Physics, Chinese Academy of Sciences, Dalian, China

**Keywords:** bufotalin, cytochrome P450 3A (CYP3A), hydroxylation, human liver microsomes (HLMs), docking simulations

## Abstract

Bufotalin (BFT), one of the naturally occurring bufodienolides, has multiple pharmacological and toxicological effects including antitumor activity and cardiotoxicity. This study aimed to character the metabolic pathway(s) of BFT and to identify the key drug metabolizing enzyme(s) responsible for hepatic metabolism of BFT in human, as well as to explore the related molecular mechanism of enzymatic selectivity. The major metabolite of BFT in human liver microsomes (HLMs) was fully identified as 5β-hydroxylbufotalin by LC-MS/MS and NMR techniques. Reaction phenotyping and chemical inhibition assays showed that CYP3A4 and CYP3A5 were key enzymes responsible for BFT 5β-hydroxylation. Kinetic analyses demonstrated that BFT 5β-hydroxylation in both HLMs and human CYP3A4 followed the biphasic kinetics, while BFT 5β-hydroxylation in CYP3A5 followed substrate inhibition kinetics. Furthermore, molecular docking simulations showed that BFT could bind on two different ligand-binding sites on both CYP3A4 and CYP3A5, which partially explained the different kinetic behaviors of BFT in CYP3A4 and CYP3A5. These findings are very helpful for elucidating the phase I metabolism of BFT in human and for deeper understanding the key interactions between CYP3A enzymes and bufadienolides, as well as for the development of bufadienolide-type drugs with improved pharmacokinetic and safety profiles.

## Introduction

Chansu, also known as toad poison or toad venom (Krenn and Kopp, 1998), has been used as an effective constituent of some well-known Chinese medicine formulas and widely used for the treatment of various diseases, including infection, pain, swelling, heart failure, as well as many types of cancer (Ye et al., 2006; Gao et al., 2010). BFT, one of the bufadienolides isolated from ChanSu, has been intensively studied due to its diverse bioactivities, such as cardiotonic, local anesthetic, blood pressure-stimulating, respiration and anticancer activities (Kamano et al., 1998; Yeh et al., 2003; Ma et al., 2009). Notably, more attention has been paid to BFT due to its dramatic anti-tumor activity, as previous structure-antitumor activity studies have showed an acetyl at C-16 position and a hydroxyl at the C-14 position could significantly enhance the anti-tumor activity of bufadienolides (Zhu et al., 2014). BFT induces apoptosis against hepatocellular carcinoma cells, where it is the most potent one among known bufadienolides, such as BF, telocinobufagin and CB (Zhang et al., 2012; Ning et al., 2015a). However, BFT and its analogs in Chansu are extremely cardiotoxic and could produce digitalis toxicity-like cardiac effects (Gowda et al., 2003). Additionally, as potent Na^+^/K^+^-ATPase inhibitors, these digoxin-like components have been shown to be involved in severe morbidity and high mortality (Mijatovic and Kiss, 2013). Therefore, investigations on the metabolic/clearance pathway(s) of BFT in humans are essential for clinical risk assessment of this toxic compound and BFT-containing TCMs.

Structurally, bufodienolides such as BF, BFT, CB, and RB possess a novel steroidal A/B *cis*, B/C *trans* and C/D *cis* ring juncture with a characteristic α-pyrone ring at C-17 position and β-hydroxyl at the C-3 position (Feng et al., 2017). Notably, BFT is an ester derivative of BF with an additional acetyl group at the C-16 position. Our previous study demonstrated that CYP3A4, the most abundant P450 isoform expressed in human liver, played a predominant role in 1β- or 5α-hydroxylations of BF, CB, and RB (Ma et al., 2011; Ge et al., 2013; Ning et al., 2015b). The isoform selectivity of CYP3A4 toward hydroxylations of these bufodienolides is very high, which is superior to the selectivity of CYP3A4 toward known steroid-type substrates, such as8 progesterone and testosterone (Zhang et al., 2008b). Unfortunately, the metabolic pathways of BFT in human tissues, as well as the effects of substituting groups at the bufodienolide scaffold on the selectivity and metabolic rates of P450 enzymes have not been well investigated.

In the present study, the phase I metabolic pathway(s) of BFT and its metabolic behaviors in human tissues was investigated for the first time. The major metabolite(s) of BFT and the key drug metabolizing enzyme(s) responsible for hepatic metabolism of BFT in human were fully characterized by a panel of standard techniques. The results demonstrated that CYP3A mediated 5β-hydroxylation is the major metabolic pathway of BFT in human liver, but the enzymatic kinetic behaviors of BFT 5β-hydroxylation in CYP3A4 and in CYP3A5 are much varied. To identify the contribution of each CYP isoform in BFT 5β-hydroxylation, as well as to explore the effects of the C-16 acyl group at the bufodienolide scaffold on the selectivity and metabolic rates of CYP3A enzymes, both experimental and computational techniques are used to explain the differential kinetic behaviors of BFT in CYP3A4 and CYP3A5. These findings are very helpful for elucidating the phase I metabolism of BFT in human, as well as for exploring the key interactions between CYP3A enzymes and bufadienolides.

## Materials and Methods

### Ethics Statement

This study was carried out in accordance with the Declaration of Helsinki. The study protocol was approved by the Ethics Committee of Peking Union Medical College (Beijing, China).

### Chemicals and Reagents

BFT and BF were purchased from Shanghai Winherb Medical Technology Company (Shanghai, China). ABT, furafylline, sulfaphenazole, clomethiazole, omeprazole, 8-methoxypsoralen, ticlopidine, CYP3cide, glucose-6-phosphate dehydrogenase, D-glucose-6-phosphate, and NADP^+^ were obtained from Sigma (St. Louis, MO, United States). Montelukast, quinidine, ketoconazole was purchased from Jianglai Biotechnology Co., Ltd. (Shanghai, China). The pooled HLMs (from 50 donors, lot no. X008067) were obtained from BioreclamationIVT (Baltimore, MD, United States). A panel of baculovirus expressed human P450s (CYP1A1, 1A2, 2A6, 2A13, 2B6, 2C8, 2C9, 2C19, 2D6, 2E1, 3A4, 3A5, 4F2, and 4F3), co-expressing NADPH-CYP reductase and cytochrome b5 were obtained from BD Gentest Corp (Woburn, MA, United States). All chemicals and solvents were of analytical grade.

### Incubation Conditions

Human liver microsomes or CYPs were incubated with NADPH-generating system, which included NADP^+^ (1 mM), glucose-6-phosphate (10 mM), glucose-6-phosphate dehydrogenase (1 unit/ml), and 4 mM MgCl_2_ in 100 mM potassium phosphate buffer (pH 7.4) in a total incubation volume of 200 μl. After a 3 min preincubation at 37°C, the reaction was initiated by the addition of NADPH-generating system and further incubated at 37°C for 30 min. The reaction was quenched with 100 μl of ice-cold acetonitrile. The samples were chilled, spun at 20,000 × g for 20 min at 4°C. Aliquots of supernatants were then stored at -20°C until analysis. All incubations throughout the study were done in three experiments conducted in duplicate with S.D. values generally below 10%.

### Analytical Instruments and Conditions

The samples were analyzed by means of the UFLC system, which equipped with an SIL-20ACHT auto sampler, a CBM-20A communications bus module, a DGU-20A3 in-line degasser, a CTO-20AC column oven, two LC-20AD pumps and an SPD-M20A photodiode array detector. BFT and its metabolites were separated by using a Shim-pack XR-ODS (75 mm × 2.0 mm, 2.2 μm, Shimadzu) analytical column with an ODS guard column (5 mm × 2.0 mm, 2.2 μm, Shimadzu). The mobile phase was comprised of CH_3_CN (A) and 0.2% formic acid (B), and the gradient profile was as follow: 0–2 min, 90–58% B; 2–8 min, 58–38% B; 8–10 min, 5% B; 10–13 min, balanced to 90% B. BFT and its metabolites (5-HBFT) were detected at 300 nm and quantified in accordance with the standard calibration curves.

Bufotalin and its metabolites were identified by using a Shimadzu LC-MS-2010EV (Kyoto, Japan) instrument with an ESI interface. With regard to mass detection, positive-ion mode (ESI+) as well as negative ion mode (ESI-) from m/z 100–800 with the electron voltage setting at +1.55 kV, and -1.55 kV was employed. Data processing was conducted using the LC-MS Solution (version 3.41; Shimadzu).

### Biosynthesis and Characterization of BFT Metabolite

The major metabolite of BFT was biosynthesized using liver microsomes from mouse (MLM, 90%) and human (HLM, 10%) with respect to the incubation system up to 250 mL. BFT (200 μM) was incubated with the liver microsomes (1.0 mg/mL) supplemented with the NADPH-generating system for 4 h at 37°C. Under these conditions, approximately 40% of BFT was converted into 5-HBFT. Then, BFT and its metabolite were separated by the HPLC (SHIMADZU, Kyoto, Japan) with a C18 column (4.6 mm × 150 mm, 10 μm). The mobile phase was 65% methanol in water. The eluent was monitored at 300 nm with a flow rate of 1.5 mL/min, and the fractions containing 5-HBFT were collected and eluted in vacuo. The purity of metabolite was above 98% according to HPLC-UV analysis. NMR spectra were carried on a Varian INOVA-400 NMR spectrometer (Varian, United States). The purified metabolite was dissolved in CDCl_3_ (Euriso-Top, Saint-Aubin, France)_,_ and chemical shifts were reported on δ scale with reference to tetramethylsilane (TMS) at 0 ppm for ^1^H-NMR (400 MHz) and ^13^C-NMR (100 MHz).

### Reaction Phenotyping Assays With Recombinant CYPs

Fifteen cDNA-expressed human P450 isoforms co-expressing NADPH-P450 reductase were used for assay of BFT hydroxylation activity. Each of the recombinant CYPs (40–80 nM) were incubated with 3 and 100 μM substrate concentrations (approximate concentration at *V*_max_, and *K*_m_ values for HLM, respectively) at 37°C for 30 min.

### Chemical Inhibition Assays

5β-hydroxylation of BFT in pooled HLM was carried out by adding selective inhibitors. In brief, BFT (10 μM, relevant to the *K*_m_ values) was incubated with or without known CYP isoform-specific inhibitors using a protein concentration of 0.25 mg protein/ml in HLM with an NADPH-generating system. The selective inhibitors and their concentrations were referred to the previous studies (Balani et al., 2002; Bjornsson et al., 2003; Feng et al., 2014).

### Kinetics Analyses

The formation rates of 5-HBFT was within the linear range of the incubation time and protein concentration. The required concentrations of BFT ranged from 10 to 250 μM. BFT was incubated with pooled HLM (0.25 mg protein/mL) for 30 min, or with recombinant CYP3A4 (5 nM) and CYP3A5 (10 nM) for 30 min. The apparent *K*_m_ and *V*_max_ values were determined by fitting experimental data to the Biphasic Kinetics equation (Eq. 1) and Substrate Inhibition equation (Eq. 2). The kinetic constants were calculated with GraphPad Prism software, version 6.0, and the results were graphically represented by Eadie–Hofstee plots.

(1) $$v=\frac{V_{\max\;1}[S]}{K_{\max\;1}+[S]}+\frac{V_{\max\;2}[S]}{K_{\max\;2}+[S]}$$

where *v* is the rate of the reaction, [*S*] is the substrate concentration, *V*_max_ is the maximum velocity estimate, *K*_m_ is the substrate affinity constant.

(2) $$v=\frac{V_{\max}}{1+(K_{\text{m}}/[S])+([S]/K_{\text{si}})}$$

where *K*_si_ is the substrate inhibition constant.

### Correlation Studies

The formation rates of the metabolite(s) described for BFT (50 μM) were measured in a panel of 12 HLMs from individuals after 15–60 min incubation in HLM (0.25 mg protein/ml). These values were correlated with the levels of CYP3A4 or CYP3A5 as well as the catalytic activities of CYP3A4 to their marker substrates. BF was an isoform-specific probe substrate, which was first reported by our laboratory and its structure was similar with BFT. BF and its hydroxylation product were determined by LC-UV at 300 nm. The concentrations of CYP3A4 and CYP3A5 in HLMs were determined using MRM mode and SIL as the reference standards. Specific peptides of EVTNFLR (for CYP3A4) and SLGPVGFMK (for CYP3A5) were selected for their quantification by using transition ion of 439.7/549.3 and *m/z* 468.3/678.5, respectively (Liu et al., 2014). The correlation coefficient was expressed by the linear regression coefficient (*R*^2^). *P* < 0.005 was considered statistically significant.

### Molecular Docking Simulations

Docking simulations were performed by using SYBYL (X-1.1) (Tripos, St. Louis, MO, United States) to generate active conformations by exploring all calculations of binding interactions and conformations of ligands. Here, the X-ray crystallographic structure of CYP3A4 ((PDB ID:3TJS) and CYP3A5 (PDB ID:5VEU) were selected for docking analysis (Sevrioukova and Poulos, 2012; Hsu et al., 2018). A low-energy conformation with no steric clashes between side chains was energy minimized for 10,000 steps with the backbone and variable modeling. Surflex-Dock used an empirical scoring function and a patented search engine to generate the bioactive binding poses of substrates in the active site of CYP3A4 and CYP3A5. During the docking simulation, the enzyme structure was kept rigid, while the substrate was left fully flexible by changing the conformations of the ligand in the active site. With the crystal structure of 3A4 and 3A5, a total of 50 bioactive binding conformations of BFT were generated by means of Surflex-Dock, which were evaluated by an empirical function of ChemScore (Ai et al., 2010). The best bioactive poses selected from the top ten solutions were then used for the analysis of the binding interactions between CYP3A and their substrates.

## Results

### Biotransformation of BFT by Human Liver Microsomes

**FIGURE 1:**
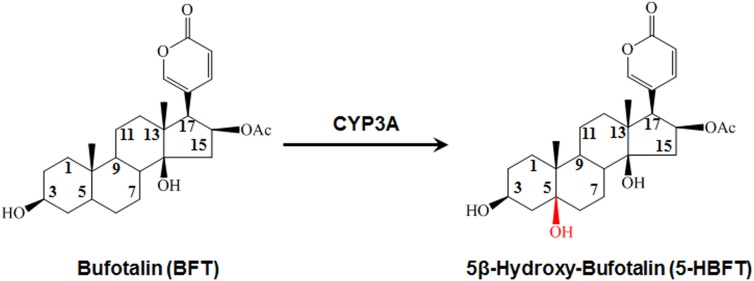
The 5β-hydroxylation of BFT in HLM.

**FIGURE 2:**
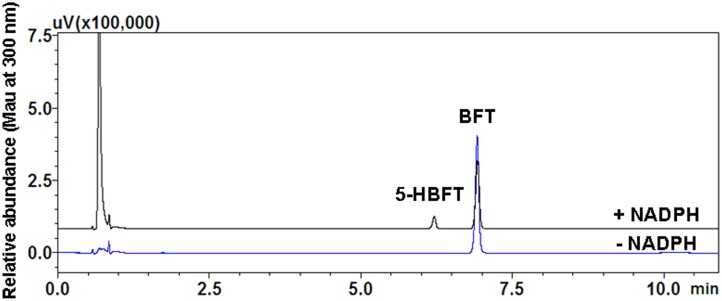
Representative UFLC-UV profile of BFT and its metabolite in HLM.

A single metabolite of BFT was identified following incubation with HLM (0.3 mg protein/ml) in the presence of the NADPH-generating system (Figure 1). As shown in Figure 2, one major product peak was eluted at 6.2 min. The metabolite was characterized by UFLC-DAD-ESI-MS, and the formation of monohydroxylbufotalin was NADPH-, time-, and microsome-dependent. The material balance data has also demonstrated the uniqueness of this metabolite (Supplementary Figure S2 and Supplementary Table S1). The [M+H]^+^ and [M+CH_3_CN+H]^+^ ion of the metabolite at m/z in HLM were 461.3 and 502.4, respectively. The molecular weight (MW) of metabolite was correspondingly calculated as 460, suggesting that this metabolite was a monohydroxylated product of BFT.

### Identification of the Major Metabolite of BFT

For elucidating the metabolic labile sites of BFT, this monohydroxylated metabolite was biosynthesized and further characterized based on ^1^H-NMR and ^13^C-NMR analysis (Table 1). In comparison with ^13^C-NMR spectrum of BFT to that of this metabolite, the additional oxygenated quaternary carbon at d 73.53 (CH) was observed instead of tertiary carbon at d 35.59 (C-5). For the meantime, three carbon signals (C-4, C-6, and C-10) presented corresponding shifts (d 33.30–d 36.98, d 26.40–d 35.14, and d 35.60–d 40.76, respectively). The upfield shifts of C-1 (Dd-64.04) and C-19 (Dd-76.08) were observed owing to the g-gauche effect, indicating that hydroxylation occurred at the C-5 site. Moreover, the NMR data of metabolite were consistent with the previously reported spectral data of marinobufagenin (Zhang et al., 2011). Based on above findings, the hydroxylated metabolite was fully identified as 5β-hydroxylbufotalin.

**Table 1:** Proton and carbon NMR chemical shift assignments for BFT and its metabolite.

Position	5β-Hydroxy-Bufotalin		Bufotalin	
	δ1H Mult (J in Hz)	δ13C	δ1H Mult (J in Hz)	Δ13C
1	1.16	23.6	1.19	29.6
	1.94		1.91	
2	1.38	24.8	1.28	27.9
	1.83		1.59	
3	4.20	68.1	4.14	66.7
4	1.56	36.9	1.24	33.3
	2.11		1.76	
5	–	73.5	1.59	35.9
6	1.47	35.1	1.46	26.4
	1.72		1.79	
7	1.28	21.5	1.32	21.1
	1.47		1.43	
8	1.50	39.1	1.49	42.3
9	1.57	41.4	1.56	35.3
10	1.83	40.7	1.84	35.6
11	1.61	28.7	1.54	21.1
	–		1.36	
12	1.25	40.5	1.39	40.9
	1.59		1.50	
13	–	49.3	–	49.4
14	–	84.4	–	84.4
15	2.85	57.1	1.53	40.4
			2.65	
16	5.52	74.4	5.54	73.6
17	2.59	40.8	2.87	57.2
18	0.79	16.4	0.79	16.4
19	0.95	16.7	0.95	23.7
20	–	116.7	–	116.9
21	7.25	151.1	7.25	151.0
22	8.00	149.0	8.03	149.2
23	6.19	113.2	6.19	113.1
24	–	161.9	–	161.9
25	–	170.0	–	170.0
26	1.87	20.9	1.87	20.9

*All spectra were recorded on a Bruker ARX-600 spectrometer, in CDCl_3_.*

### Reaction Phenotyping Assays

**FIGURE 3:**
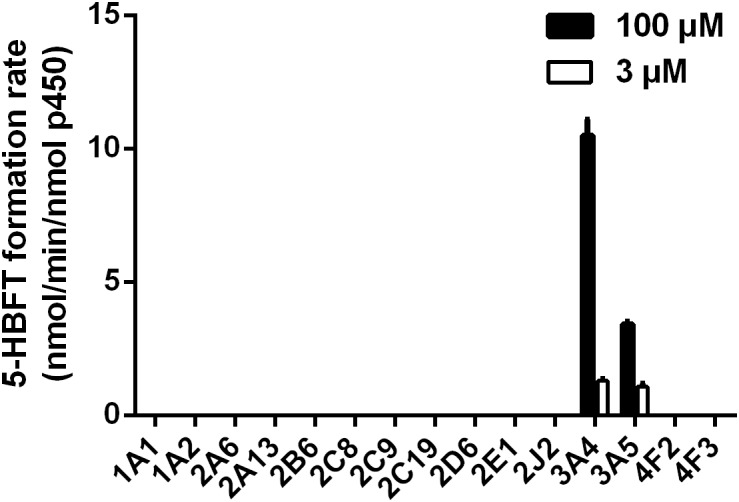
Reaction phenotyping assays of BFT hydroxylation by using a panel of human CYPs.

In order to explore the involved P450 isoform(s) for the metabolism of BFT in humans, the formation of 5-HBFT was evaluated using a series of P450 isoforms. As shown in Figure 3, one hydroxylated metabolite was mediated specifically by CYP3A4 and CYP3A5, while no metabolites were detected in the incubation systems with other P450 isoforms. The formation rates of 5-HBFT in CYP3A4 were 1.30 ± 0.05 and 10.50 ± 0.42 per min/nmol P450 upon addition of BFT with the substrate concentrations of 3 and 100 μM, respectively. In contrast, the BFT 5β-hydroxylation rates in CYP3A5 were relatively slow, with the 5-HBFT formation rates were 1.08 ± 0.08 and 3.42 ± 0.07 per min/nmol P450, respectively.

### Chemical Inhibition Assays

**FIGURE 4:**
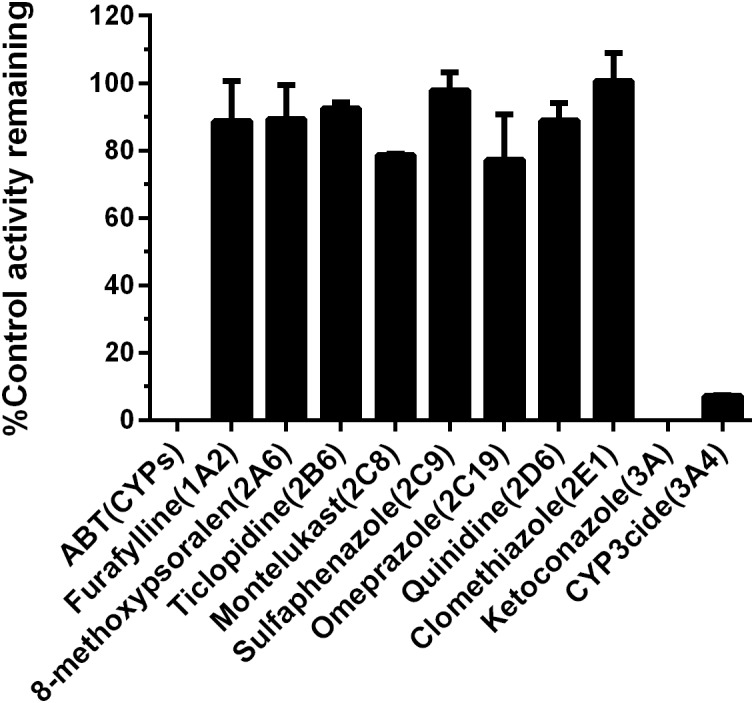
Chemical inhibition assays of BFT 5β-hydroxylation by selective CYP inhibitors in HLM.

To further verify the key P450 isoform(s) involved in the formation of 5-HBFT in HLM, chemical inhibition assays were conducted by using a panel of selective inhibitors of major P450 isoforms. As shown in Figure 4, ABT (a broad P450 inhibitor) could inhibit the formation of 5-HBFT completely, indicating that BFT hydroxylation was highly P450-specifc. Among all tested isoform-selective inhibitors, ketoconazole (CYP3A inactivator) completely inhibited the catalytic activity of HLM, further indicating CYP3A played the conspicuous role in BFT hydroxylation. Moreover, CYP3cide, an exclusive inhibitor of CYP3A4, could inhibit ∼90% formation of 5β-hydroxlbufatalin in HLM, suggesting that CYP3A4 is the predominant enzyme accountable for BFT 5β-hydroxylation. In contrast, the selective inhibitors against other P450 isoforms did not show significant inhibitory effects (less than 25% inhibition, *P* > 0.05) on this biotransformation. These results suggested that BFT was selectively metabolized by CYP3A, while CYP3A4 play a key role in the formation of 5β-hydroxlbufatalin in human liver.

### Kinetic Analyses

**FIGURE 5:**
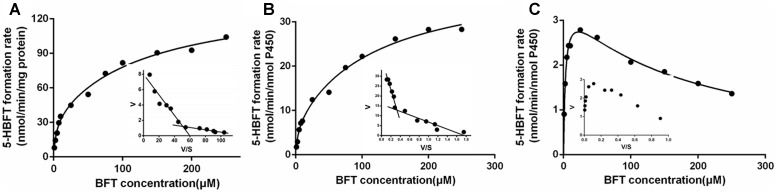
Kinetic plots of BFT 5β-hydroxylation in HLM **(A)**, CYP3A4 **(B)** and CYP3A5 **(C)**.

Over the entire concentration range examined, the kinetics behaviors of 5β-hydroxylation of BFT, in HLM and CYP3A4 followed the biphasic kinetics, but this reaction obeyed the substrate inhibition kinetic in CYP3A5, as evidenced by Eadie–Hofstee plot (Figure 5). In addition, the similar apparent kinetic parameters were observed in the kinetic characterization of BFT 5β-hydroxylation in HLM and CYP3A4. In HLM, *K*_m1_ and *K*_m2_ values for the formation of 5-HBFT were 3.71 and 133.70 μM, respectively, while the *V*_max1_ and *V*_max2_ values for 5-HBFT formation were 33.31 and 107.30 per min/mg, respectively. Similarly,the *K*_m1_ and *K*_m2_ values of BFT 5β-hydroxylation by CYP3A4 were 3.46 and 122.40 μM, respectively, whereas the values of *V*_max1_ and *V*_max2_ were 6.87 and 33.70 per min/nmol P450, respectively (Table 2). In CYP3A5, the *K*_m_ and the *K*_i_ value for the formation of 5-HBFT was 2.91 and 170.00 μM, the corresponding *V*_max_ value was 3.45 nmol/min per nanomole P450. These result implied that BFT displayed different kinetic behaviors in CYP3A4 and CYP3A5.

**Table 2:** Kinetic parameters of BFT 5β-hydroxylation in HLM, recombinant human CYP3A4 and CYP3A5.

Enzyme Source	High-affinity site		Low-affinity site	
	*V*_max_	*K*_m_	*V*_max_	*K*_m_
HLM	33.31 ± 12.64	3.71 ± 2.68	107.30 ± 11.77	133.70 ± 72.23
CYP3A4	6.87 ± 3.59	3.46 ± 3.51	33.70 ± 3.08	122.40 ± 56.5

	***V*_max_**	***K*_m_**	***Ksi***	***CL_int_***
		
CYP3A5	3.45 ± 0.09	2.91 ± 0.24	170.00 ± 12.69	1186.12

*K_m_ values are μM, V_max_ values are nmol ⋅ min^−1^ ⋅ mg^−1^ for liver microsomes or nmol ⋅ min^−1^ ⋅ nmol^−1^ of P450 for CYP3A. The range of substrate concentrations was 1–250 μM. Each value was the mean ± S.D. of three determinations performed in duplicate.*

### Correlation Studies

**FIGURE 6:**
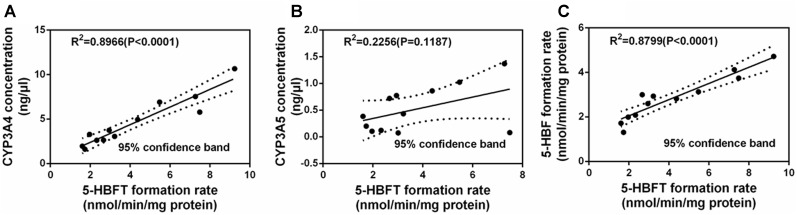
Correlation studies between the formation rates of 5-HBFT and the levels of CYP3A4 **(A)**, or the levels of CYP3A5 **(B)**, and the formation rates of 5-HBF **(C)**, in a panel of twelve HLMs from individuals.

It is well-known that CYP3A4 and CYP3A5 are two major CYP3A isoforms distributed in adult human liver, and these two enzymes share high amino acid sequence identity (>84%). Generally, the abundance of CYP3A5 is relative lower than that of CYP3A4 in human liver. Thus, in most cases, CYP3A4 has been considered to be a key determinant crucial for the hepatic metabolism of CYP3A substrates. In this study, the correlations between the expression levels of CYP3A4 or CYP3A5 expression levels and BFT hydroxylation were carefully studied to reveal which isoenzyme was the major enzyme responsible for hepatic metabolism of BFT (Daly, 2006). To this end, the determination of the formation rates of 5-HBFT were using a panel of 12 HLMs from different individual donors, while the proteins levels of CYP3A4 or CYP3A5 were also determined by a proteomics-based method. After that, the formation rates of 5-HBFT were compared with the protein levels of CYP3A4 or CYP3A5 in individual HLMs. As shown in Figure 6A,B, a strong linear regression coefficient (*R*^2^) between the formation rates of 5-HBFT and the expression levels of CYP3A4 in individual HLMs (*R*^2^ = 0.8966, *P*< 0.001) was observed, while the formation rates of 5-HBFT is poorly correlated with the CYP3A5 levels (*R*^2^ = 0.2256, *P* = 0.1187). Furthermore, the CYP3A activities in individual HLMs measured by using BFT was also compared with the CYP3A4 activities determined by BF, a previously reported isoform-specific probe substrate for CYP3A4. As shown in Figure 6C, a splendid correlation between the formation rates of 5-HBFT and the formation rates of 5-HBF was observed (*R*^2^= 0.8799, *P*< 0.001). These findings suggested that CYP3A4 played a key role in BFT 5β-hydroxylation in HLM from Mongolian Populations.

### Molecular Docking Simulations

**FIGURE 7:**
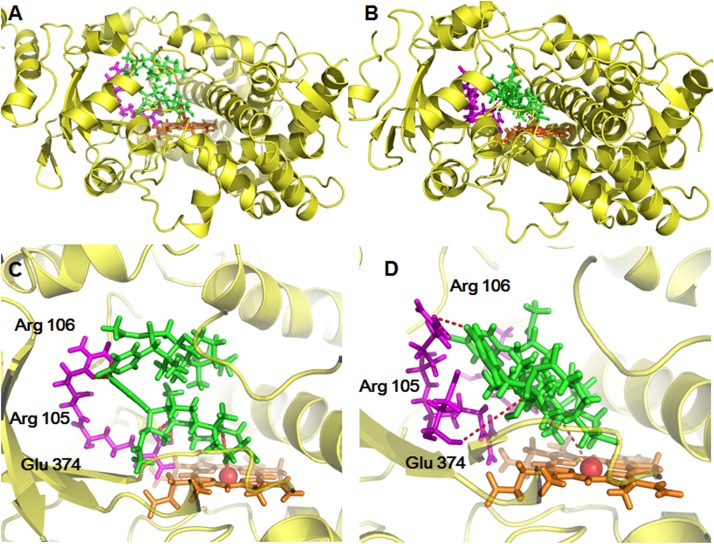
The stereo views of docking simulations of BFT into the active cavity of both CYP3A4 **(A)** and CYP3A5 **(B)**. The detailed views of the binding areas showed that BFT on CYP3A4 and CYP3A5, while BFT could docked into different ligand binding site of CYP3A4 **(C)** and CYP3A5 **(D)**.

For further providing deep insights into CYP3A-mediated BFT 5β-hydroxylation, molecular modeling simulations were performed from the perspective of recognition and binding between the substrate and two CYP3A enzymes. It is well-known that the active cavity of both CYP3A4 and CYP3A5 are relatively large and flexible, both enzymes have been identified with more than two different ligand-binding sites (Isin and Guengerich, 2006). As shown in Figure 7, the active cavity of both CYP3A4 and CYP3A5 could accommodate two molecular substrates (BFT). In CYP3A4, two BFT molecules are stacked in a parallel orientation, the first one (BFT) could be well-docked into the catalytic site with the H atom of the C-5 site facing the heme iron at 4.35 Å, while the second one could bind on the surrounding area near to the first one with relatively far site-heme distance (11.00 Å). The closer BFT to the heme in the model will be termed active BFT, while the other BFT molecule will be termed effector BFT. The results indicated that the active BFT would facilitate the subsequent binding effector BFT. In contrast, the binding areas of two molecules of BFT in CYP3A5 were highly overlapped, the active BFT lied against the effector BFT molecule in the CYP3A4 active site with site-heme distance 5.49 and 7.18 Å. The presence of the effector BFT molecule shifted active BFT away from the heme, and the molecule no longer rotated freely in the active site, implying that the binding of the effector molecule would affect the binding of the active substrate in CYP3A5 (Table 3). In addition, the acetate group at the C-16 site of active BFT formed hydrogen bonds with the Glu374 of both CYP3A4 and CYP3A5, which could fix the active substrate in a favorable position for the further metabolic reaction, indicating that the acetate group may play a critical role in positioning the active substrates in recognition and binding of CYP3A enzymes. Moreover, Glu374 seemed to be important for CYP3A specificity (Figure 7C,D). These findings agreed well with the experimental data and could partly explain why BFT 5β-hydroxylation displayed the autoactivation kinetics in CYP3A4, while in CYP3A5, obeyed the substrate inhibition kinetics.

**Table 3:** BFT 5β-hydroxylation related parameters derived from the molecular modeling of BFT with the crystal complex of CYP3A4 and CYP3A5, respectively.

Parameters	CYP3A4 (near)	CYP3A4 (far)	CYP3A5 (near)	CYP3A5 (far)
Hammerhead score	−33.70	−18.96	−26.88	−22.14
Site-heme distance	4.35 Å	11.00 Å	5.49 Å	7.18 Å

## Discussion

As a biologically active compound of the bufadienolides isolated from ChanSu, BFT has drawn increasing attentions in recent years especially for its anticancer effects. Recent studies have revealed that BFT could regulate a variety of cellular activities, such as proliferation, differentiation, apoptosis, as well as glucose metabolism, angiogenesis and multidrug resistance in human tumors (Dong et al., 2011; Salvador et al., 2013). Therefore, it is highly desirable to fully characterize the metabolic pathway(s) of BFT to expedite the process for the drugability assessment of this bufadienolide. Although the metabolic pathways of BFT in mice and in some microorganisms have been reported (Xin et al., 2009; Li et al., 2011; Huang et al., 2015), the metabolic pathways of BFT in human tissues have not been reported yet. In this study, the metabolite profile of BFT in HLMs has been well studied and the major metabolite and the involved metabolic enzymes have been identified for the first time. Our results clearly demonstrated that BFT could be readily catalyzed by CYP3A4 and CYP3A5 to 5-HBFT in HLM, which is consistent with the metabolic pathway(s) of BFT in mice. However, the catalytic efficacy and the kinetic behaviors of BFT 5β-hydroxylation are quite different in CYP3A4 and CYP3A5. Taking into account that CYP3A enzymes are the predominant contributors in hepatic metabolism of BFT, the individual discrepancy in BFT metabolism may be very significant due to the large inter-individual differences in the expression and function of CYP3A (Liu et al., 2017). Although the levels of CYP3A4 in liver is generally higher than CYP3A5, but in some cases, the levels of CYP3A5 in given populations (such as African) may higher than that of CYP3A4 (Westlind-Johnsson et al., 2003; Lu et al., 2012). Given that the relative content of CYP3A5 to total hepatic CYP3A protein varies remarkably among individuals (17–50%), the contribution of CYP3A5 in BFT metabolism and the kinetic behaviors of BFT 5β-hydroxylation may be strongly affected by the expression and function of CYP3A5 (Wu et al., 2016).

Our previous studies have found that CYP3A4 played a leading role in hydroxylations of bufadienolides (Supplementary Figures S1, S3) (Ma et al., 2011; Ge et al., 2013). However, the present study demonstrated that the increased contribution of CYP3A5 was involved in BFT 5β-hydroxylation in comparison with other naturally occurring bufadienolides. Docking simulations demonstrated that the distance between the H atom of the C-5 site and the heme of CYP3A4 of BF was shorter than that in CYP3A5, while the bioactive pose of BF in 3A4 given higher hammerhead score values than that in 3A5. These findings agreed well with the experimental results, in which BF 5β-hydroxylation catalyzed by CYP3A4 was easier than that by CYP3A5 (Supplementary Figure S4 and Supplementary Table S2). The improved CYP3A5 preference could be attributed to their subtle structural differences at the C-16 site. From the views of the chemical structure, the acetyl substituent group has been introduced into the specific carbon of bufadienolides, which may affect the isoform selectivity and the kinetics behaviors of 5β-hydroxylation in both CYP3A4 and CYP3A5. It was apparent from Figure 4 and Table 2 that BFT 5β-hydroxylation in HLM and CYP3A4 followed the biphasic kinetics, while in CYP3A5, obeyed the substrate inhibition kinetics, which differed from BF formed mono-hydroxylated metabolites and followed Michaelis–Menten kinetics in CYP3A4 (Supplementary Table S3). These results suggest that the presence of acetyl substituent group at C-16 site may affect the binding and catalytic process between BFT and CYP3A4 and CYP3A5. It has been reported CYP3A subfamily, which has large substrate-binding pockets, is competent to bind two or more molecules of same substrates that exhibit homotropic or heterotropic cooperativity (Yusuke et al., 2008; Zhang et al., 2008a; Davydov et al., 2012; Raunio et al., 2015). Both kinetic analyses and docking simulations demonstrated that the introduction of the acetyl substituent group, a hydrophilic radical would contribute to BFT bind with two different ligand binding site of both CYP3A4 and CYP3A5, especially through the formation of H-bond with the residual Glu374, which then led to different allosteric effects (Cameron et al., 2005; Sohl et al., 2008). The binding of active BFT molecule (the closer BFT to the heme) to CYP3A4 was deemed to make effector BFT restrain in a stacked parallel configuration, which triggered a structural transition that facilitated the succedent binding event(s), whereas an opposite structural transition that slowed down the progress of binding event(s) when BFT molecule bound to CYP3A5, the presence of the effector BFT molecule shifted active BFT away from the heme, in turn resulting in different catalytically competent enzyme-substrate complexes.

The different kinetic behaviors of BFT-hydroxylation in both CYP3A4 and CYP3A5 could also be attributed to the distinctions of the active site of CYP3A4 and CYP3A5. Although the secondary and tertiary structures of CYP3A5 have a strong resemblance of CYP3A4, the CYP3A5 active site is relatively taller and narrower than that of CYP3A4, which may affect the substrate-binding process (Emoto and Iwasaki, 2006; Hsu et al., 2018). Previous study indicated that the narrow cavity and position of Glu374 probably contribute to the higher selectivity of 3A5 over 3A4 for the *O*-demethylation of schisantherin E (Wu et al., 2017). In present study, the docking study also verified that the binding of the active BFT to the narrower active site of CYP3A5 triggered contractions through the hydrogen bonds with the Glu374, which made the binding areas of two molecules of BFT highly overlap. By contrast in CYP3A4, the active BFT could be well-docked into the catalytic site, which expanded the active site cavity that enabled the effector BFT to bind on the surrounding area near to the active molecule. Therefore, these findings might be able to explain why BFT 5β-hydroxylation displayed the autoactivation kinetics in CYP3A4, while in CYP3A5, obeyed the substrate inhibition kinetics.

The structure-activity relationship studies revealed biotransformation of bufadienolides could remarkably alter the cytotoxic activities, especially hydroxylated products showed even more potent activity than their precursors against cancer cell (Ye et al., 2004). Our previous studies demonstrated that CYP3A-mediated RB 5β-hydroxylation in humans produces the bioactive metabolite marinobufagenin with superior metabolic stability, implying that RB might be useful as a prodrug whose anticancer effects *in vivo* could still be preserved after biotransformation (Ning et al., 2015b). Likewise, 5β-hydroxyl BFT may show similar or even more potent cytotoxicities comparable to BFT. Recent studies demonstrated that all metabolites achieved similar maximum tumor inhibition rates as BFT regardless of hydroxylation or dehydrogenation, which suggested BFT could be considered as a potential leading anticancer agent (Han et al., 2016). Therefore, BFT presents a promising antitumor potential and deserves further investigation.

## Conclusion

In summary, the phase I metabolic pathways of BFT has been carefully characterized. The major metabolite of BFT in HLM was purified and fully identified as 5β-hydroxylbufotalin, while CYP3A4 and CYP3A5 were the major isoform responsible for BFT 5β-hydroxylation. BFT 5β-hydroxylation in both HLM and CYP3A4 obeyed the biphasic kinetics, while such biotransformation in CYP3A5 followed the substrate inhibition kinetics. Molecular docking simulations were also performed to explore the interactions between BFT and CYP3A, and the results demonstrated that BFT could bind on two different ligand-binding sites including one high-affinity and one low affinity site on both CYP3A4 and CYP3A5, which was very different from other natural occurring bufadienolides including BF and CB. All these findings suggested that CYP3A-mediated 5β-hydroxylation was the major metabolic pathway of BFT in the human liver, while the acyl substituent group at C-16 site of bufadienolides could affect the catalytic behaviors and enzyme specificity of bufadienolides.

## Author Contributions

Z-RD and JN were involved in the project design, carried out most of the experiments, and drafted the manuscript. G-BS, J-JW, H-YM, and FZ contributed to the data analysis. PW, L-WZ, and JH participated in the biosynthesis and characterization of metabolite. G-BG, X-BS and LY designed and supervised this study. All authors read and approved the manuscript finally.

## Conflict of Interest Statement

The authors declare that the research was conducted in the absence of any commercial or financial relationships that could be construed as a potential conflict of interest.

## Abbreviations

5-HBFT: 5β-hydroxylbufotalin
ABT: 1-aminobenzotriazole
APM: advanced protein modeling
BF: bufalin
BFT: bufotalin
CB: cinobufagin
*CL*_int_: intrinsic clearance
CYP: cytochrome P450
ESI: electrospray ionization
HLM: human liver microsomes
*K*_m_: apparent affinity
NADPH: nicotinamide-adenine dinucleotide phosphate
NMR: nuclear magnetic resonance
RB: resibufogenin
TCM: traditional Chinese medicine
UFLC-DAD: ultra-fast liquid chromatography-diode array detector
*V*_max_: apparent maximum reaction velocity.
