# Supplementary material for: Cytochrome P450 3A Enzymes Are Key Contributors for Hepatic Metabolism of Bufotalin, a Natural Constitute in Chinese Medicine Chansu
Source: Front Pharmacol. 2019 Feb 4;10:52. doi: 10.3389/fphar.2019.00052 (PMC6369212; doi:10.3389/fphar.2019.00052)
Supplement: Supplementary file 1 [file Table_1.doc]

Supplementary materials

for

**Cytochrome P450 3A enzymes are key contributors for hepatic metabolism of bufotalin, a natural constitute in Chinese medicine Chansu**

Zi-Ru Dai ^a,1^, Jing Ning ^b,1^, Gui-Bo Sun ^a^, Ping Wang ^c^, Feng Zhang ^c^, Hong-Ying Ma ^c^, Li-Wei Zou ^c^, Jie Hou ^b^, Jing-Jing Wu ^d^, Guang-Bo Ge ^c,d,*^, Xiao-Bo Sun ^a, *^, Ling Yang ^c^

^a^ Key Laboratory of Bioactive Substances and Resources Utilization of Chinese Herbal Medicine, Ministry of Education, Institute of Medicinal Plant Development, Chinese Academy of Medical Sciences & Peking Union Medical College, Beijing, 100193, China.

^b^ College of Pharmacy, Dalian Medical University, Dalian, 116044, China.

^c^ Institute of Interdisciplinary Integrative Medicine Research, Shanghai University of Traditional Chinese Medicine, Shanghai, 201203, China.

^d^ Dalian Institute of Chemical Physics, Chinese Academy of Sciences, Dalian, 116023, China.

*Corresponding authors. Tel.: +86 21 51323184; fax: +86 21 51323184.

E-mail addresses: sun_xiaobo@163.com (X.-B. Sun) & geguangbo@dicp.ac.cn (G-B. Ge)

^1^ These authors contributed equally to this work.

**Contents:**

1.Table S1 Material balance data of BFT and its metabolite in HLM---------------------------3

2.Table S2 BF 5β-hydroxylation related parameters derived from the molecular modeling of BF with the crystal complex of CYP3A4 and CYP3A5, respectively-----------------------3

3.Table S3 Kinetic parameters of BF 5β-hydroxylation determined in different enzyme resources ------------------------------------------------------------------------------------------------3

4. Fig. S1 Chemical structures of BF and BFT ----------------------------------------------------4

5. Fig. S2 UFLC-UV profile of BFT and its metabolite in HLM -------------------------------4

6. Fig. S3 Isozyme specificity of BF 5β-hydroxylation ------------------------------------------5

7. Fig. S4 The stereo views of docking simulations of BF into the active cavity of both CYP3A4 (A) and CYP3A5 (B). The detailed views of the binding areas showed that BF on CYP3A4 (C) and CYP3A5 (D). ------------------------------------------------------------------- 5

**Table S1** Material balance data of BFT and its metabolite in HLM.

| **Compound (%)**  **Time (min)** | **5-HBFT** | **BFT** | **Sum** |
| --- | --- | --- | --- |
| **0** | 0 | 99.5 | 99.5 |
| **30** | 11.1392 | 88.0247 | 99.1639 |
| **60** | 24.5928 | 73.5708 | 98.1636 |

**Table S2** BF 5β-hydroxylation related parameters derived from the molecular modeling of BF with the crystal complex of CYP3A4 and CYP3A5, respectively.

| **Compound** | **BF** | |
| --- | --- | --- |
| **Parameters** | **CYP3A4** | **CYP3A5** |
| Hammerhead score | -35.46 | -28.96 |
| Site-heme distance | 4.05 Å | 5.46 Å |

**Table S3** Kinetic parameters of BF 5β-hydroxylation determined in different enzyme resources.

| **Enzyme Source** | ***V_max_*** | ***K_m_*** | ***V_max_ /K_m_*** |
| --- | --- | --- | --- |
| HLMs  CYP3A4 | 2.62 ± 0.04  17.33 ± 0.29 | 8.00 ± 0.52  10.17 ± 0.67 | 327.50  1704.03 |
| CYP3A5 | 0.25± 0.02 | >150 | <1.67 |

***K_m_*** values are μM, ***V_max_*** values were in nmol/min/mg protein for liver microsomes, or in nmol/min/nmol CYP for CYP3A. The range of substrate concentrations was 1 to 150 μM. Each value was the mean ± S.D. of three determinations performed in duplicate.

.**
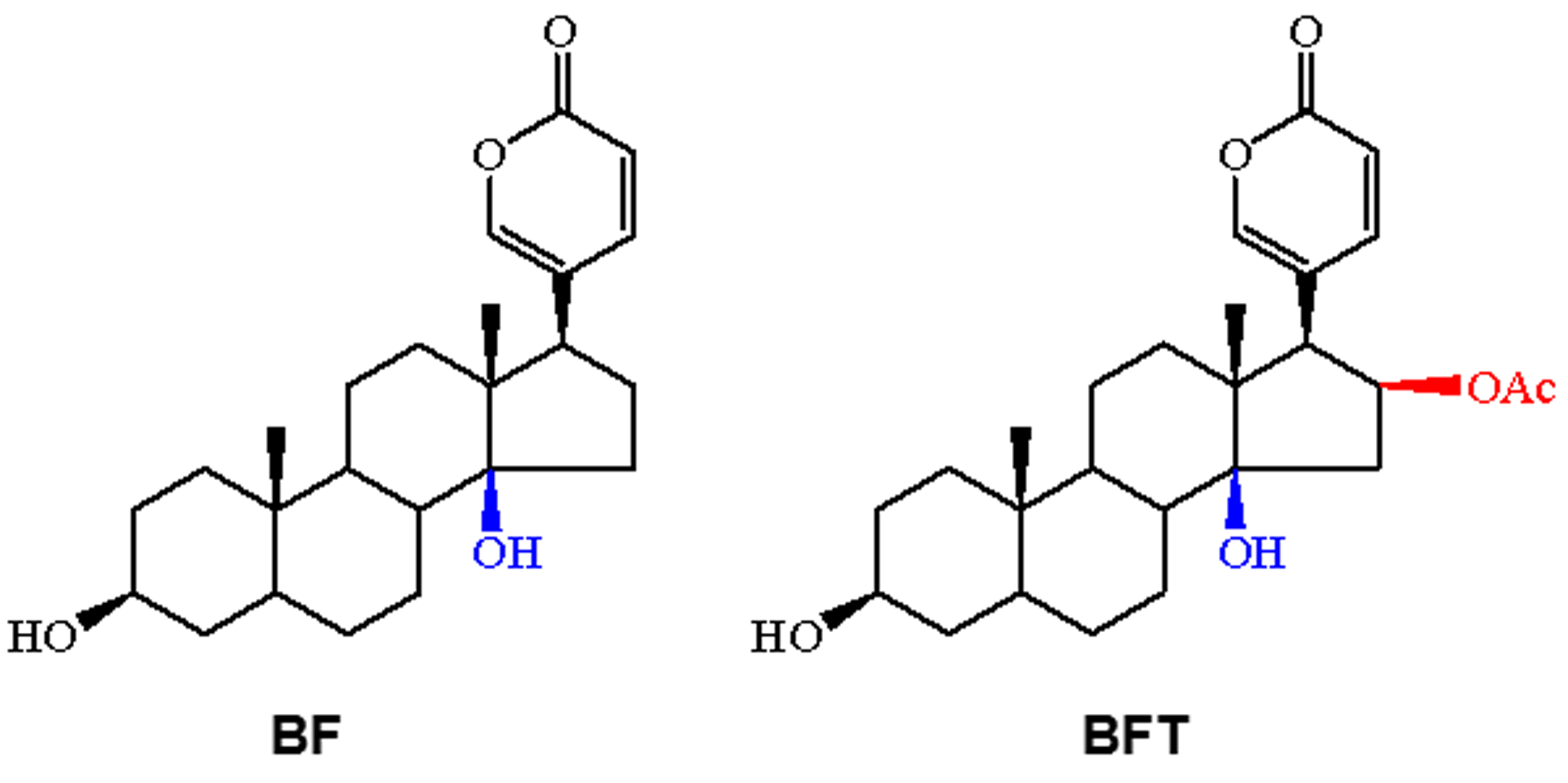
**

**Fig. S1** Chemical structures of BF and BFT.


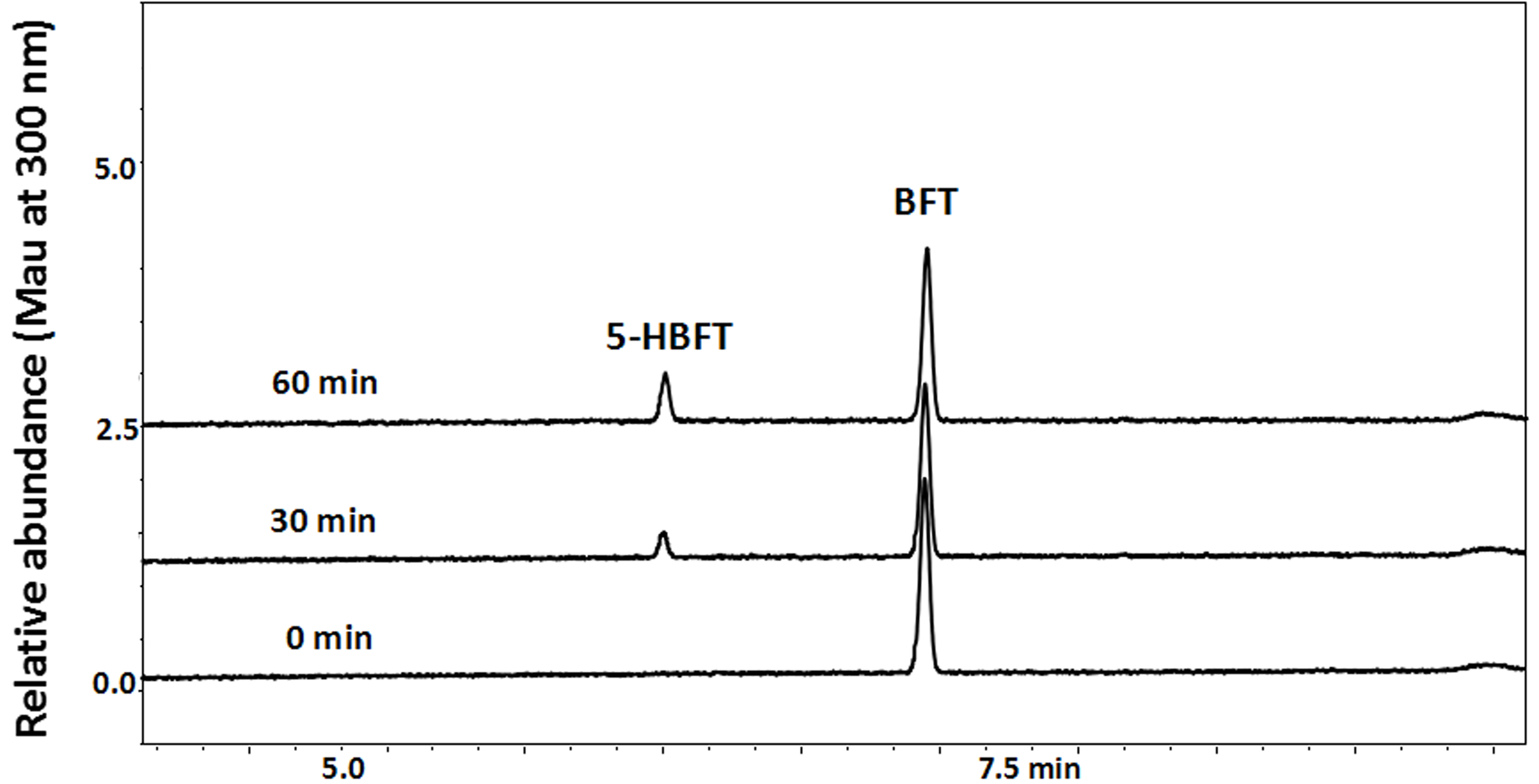


**Fig. S2** UFLC-UV profile of BFT and its metabolite in HLM.





**Fig. S3** Isozyme specificity of BF (100 μM) 5β-hydroxylation.


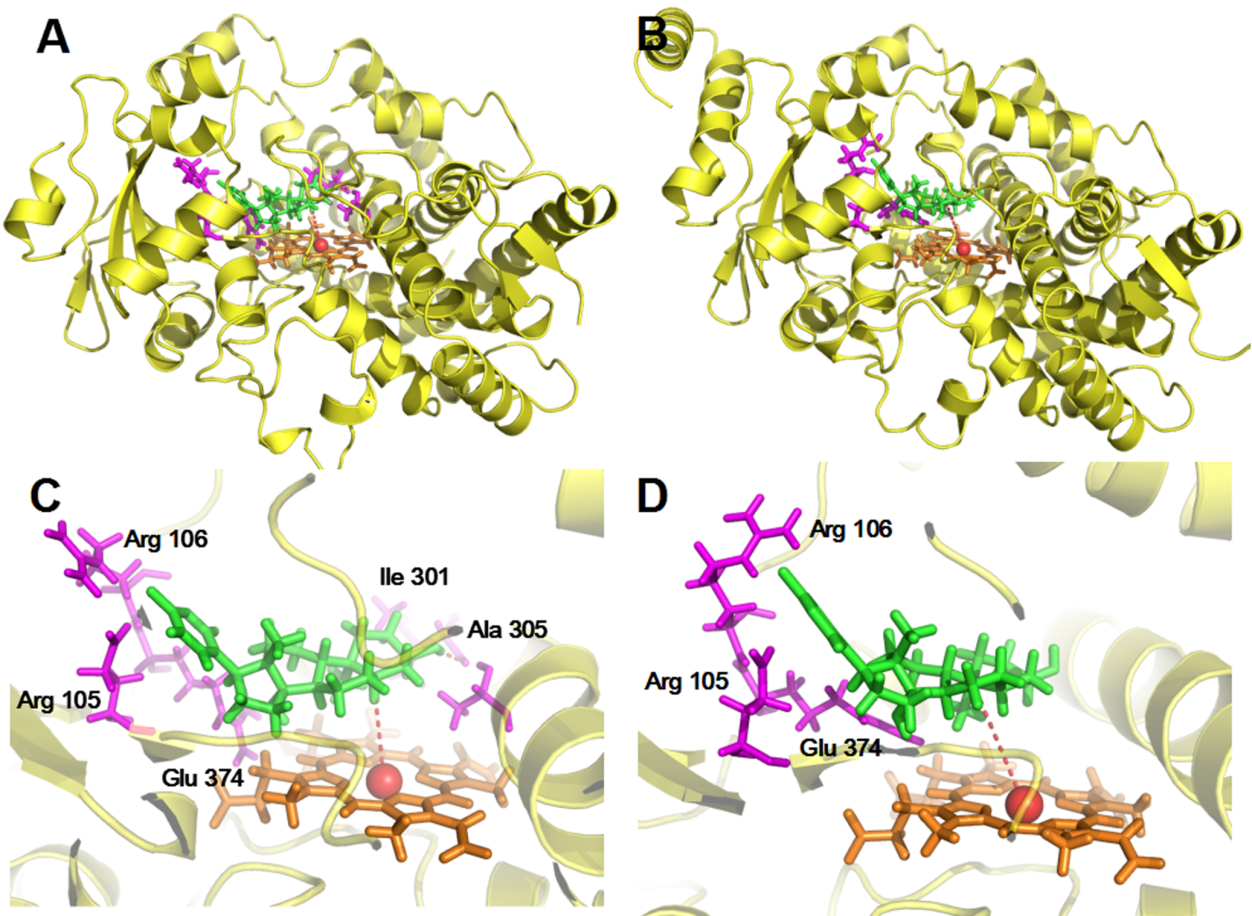


**Fig. S4** The stereo views of docking simulations of BF into the active cavity of both CYP3A4 (A) and CYP3A5 (B). The detailed views of the binding areas showed that BF on CYP3A4 (C) and CYP3A5 (D).
